# Integrating Acceptance and Commitment Therapy (ACT) with Psychological Skills Training (PST): a case study

**DOI:** 10.3389/fpsyg.2025.1617548

**Published:** 2025-07-29

**Authors:** Samuel Wood, Martin James Turner

**Affiliations:** ^1^Department of Sport and Exercise Sciences, Manchester Metropolitan University Institute of Sport, Manchester, United Kingdom; ^2^Department of Psychology, Manchester Metropolitan University, Manchester, United Kingdom

**Keywords:** CBT, MST, performance, sport psychology, psychotherapy, youth

## Abstract

This case study outlines the sport psychology service delivery provided to an 11-year-old competitive figure skater. The client reported performance anxiety, which hindered her training and performance at competition. The intervention delivered integrated core processes of acceptance and commitment therapy (ACT) with psychological skills training (PST) with the aim of reducing the client’s performance anxiety. Little has been written about how ACT can be applied alongside PST. The case reports how ACT exercises aimed at defusing the client’s unhelpful cognitions, focusing on valued living, committed action, and staying in the present moment were integrated into goal setting, imagery, performance profiling, and self-talk. Demonstrating the alignment between ACT and PST is crucial for practitioners to seamlessly integrate ACT into their practice. Reflections from the client and practitioner capture the evaluation of the service delivery.

## Introduction

Arguably, the demands placed on children in the youth-sport setting are enormous given their limited cognitive, social, and emotional capacity (Thompson and Hilliard, 2021). This has led to youth athletes becoming a growing client base for applied sport psychologists (Thrower S. et al., 2024). Importantly, developing key mental skills early in the careers of youth athletes eliminates their reliance on “quick fixes” when burnout, fear of failure, or anxiety occur (MacNamara and Collins, 2015). This results in the regular and proficient use of specific mental techniques (Woodcock et al., 2011), and the development of important mental qualities (e.g., self-confidence; Holland et al., 2010). This case study details an intervention delivered in applied practice to a youth athlete experiencing performance anxiety.

Performance anxiety is typically viewed as a reaction to the perceived stress of performing under pressure (Ford et al., 2017). Although various definitions of sport anxiety exist (see Patel et al., 2010), anxiety may present behaviourally (e.g., withdrawal from social support, or avoiding certain settings), be a stable part of an individual’s personality (i.e., trait anxiety), or be situation-specific (i.e., state anxiety). Anxiety has been categorised into cognitive (e.g., negative thoughts and inattention) and somatic (e.g., sweating and elevated heart rate) components. Importantly, when anxiety is left unchecked it can lead to clinically significant psychological disturbances and be detrimental to athletes’ wellbeing and performance (Ford et al., 2017).

Practitioners in applied sport, exercise, and performance (SEP) domains should be aware of how anxiety relates to individual’s cognitive appraisals, physiological arousal, and performance. Several relationships between sport performance and anxiety have been hypothesised, with the inverted U (Yerkes and Dodson, 1908) – the idea that that increased anxiety is facilitative to a certain point and then becomes debilitative – perhaps the most influential. Hardy and Frazey (1987) catastrophe model argues that although anxiety can gradually facilitate performance, performance can deteriorate steeply when anxiety becomes overwhelming. Hanin’s (2007) Individual Zone of Optimal Functioning (IZOF) recognises the interpretation of athlete attributes to their arousal as an important correlate to performance, distinguishing between functional and dysfunctional emotion intensity.

## Assumptions of practice

A first-person writing style is employed throughout to aid a personal and comprehensive written account of the intervention delivered. Based in the United Kingdom, I (first author) am a British Psychological Society (BPS) Chartered Sport Psychologist and a Registered Practitioner Psychologist with the Health and Care Professions Council (HCPC). My applied experiences include consulting on an individual basis with clients from various sports (e.g., swimming, golf, and figure skating) and various ages (e.g., youth and adult) over a 7-year period. The second author, also a HCPC Registered Practitioner, actively contributed to the writing and editing of this case for publication.

Through completion of the BPS independent training route for Chartered Psychologist status, I explored various psychotherapy approaches to service delivery. Reflecting on my personal values (Cropley et al., 2007), I gravitated towards an interpretivist and constructivist philosophy to consultancy (Keegan, 2016). Taking a client-led approach, and the belief that the client is the expert of their situation (Rogers, 1977), I favoured service delivery tailored to the client’s needs. Acknowledging the growing body of literature highlighting that applied sport psychology is not limited to psychological skills training (PST) alone (see Wedding and Corsini, 2019), I had learned of the interplay between thoughts, feelings, behaviours, and physiology, and the use of strategies to challenge or control unhelpful internal states that impact performance (Turner et al., 2023). The range of psychotherapeutic approaches that form theoretical orientations for practice delivery was overwhelming to navigate (Tod and Eubank, 2020). Over time, I aligned with the psychotherapeutic processes of acceptance and commitment therapy (ACT) – a so called third-wave cognitive behavioural approach, which is increasingly applied to performance settings (Juncos and de Paiva e Pona, 2018) including sport (see Wood and Turner, 2025b) – appreciating that controlling internal states might worsen the presenting problem(s) (Hayes et al., 1999). Developing my personal and professional philosophy (Poczwardowski et al., 2004), ACT provided a clear theoretical framework for my practice (Tod and Eubank, 2020).

### An overview of ACT

Acceptance and commitment therapy is a flexible, principles-centred approach to delivery (Ong et al., 2024) that can be applied to most presenting problems encountered in applied sport psychology (Hegarty and Huelsmann, 2020). ACT interventions aim to move the client from *psychological inflexibility* (i.e., cognitive fusion, experiential avoidance, behavioural rigidity, and inactivity) towards *psychological flexibility* (i.e., mindfulness, acceptance, and clarification of goals and values) to change overt behaviour (Harris, 2019). ACT does this through six core therapeutic processes that are depicted within the ACT hexaflex. There is no correct order to consider these.

*Defusion* is a defining component of psychological flexibility that aims to create distance between the individual and unhelpful thoughts, reducing the power (and negative impact) unhelpful cognitions have on emotions and behaviours (Assaz et al., 2023). Although discussed as a procedure, process, and outcome, defusion might be best considered an outcome (Assaz et al., 2023).

Fusing with painful memories (e.g., rejection, disappointment, or failure) might manifest as worry, rumination, or ongoing negative commentary. This can create ongoing attempts to avoid or escape difficult thoughts (and feelings) – termed *experiential avoidance* – and increase psychological suffering (Harris, 2019). ACT encourages *acceptance* of unhelpful cognitions; viewing them with less judgement and less literally (Harris, 2019).

*Contact with the present moment* relates to our ability to pay attention to, and engage in, here-and-now experiences (Harris, 2019). Intentionally focusing on the present moment, without judgement, is more widely discussed as mindfulness (Kabat-Zinn, 2023). The opposite is *inflexible attention* – difficulty concentrating on a task; losing interest or involvement in an experience; and disconnecting from thoughts and feelings (Harris, 2019).

*Values* relate to how we want to behave, treat ourself, and others, both in a given moment and an ongoing basis (Harris, 2019). Like a compass, values provide a direction of travel. Typically, as behaviour becomes increasingly driven by fusion and experiential avoidance, values become neglected or forgotten.

*Committed action* means taking effective (physical and psychological) action to live our values (Harris, 2019). This might involve goal setting, action planning, problem solving, and skills training to adapt to the challenges of a given situation.

*Self-as-context*, one of the more complex aspects of the ACT hexaflex model (also termed the “noticing self” or “observing self”) focuses on drawing attention to our awareness so we can learn to see thoughts as thoughts (Harris, 2019).

### An overview of psychological skills training

Despite aligning with ACT, I continued to value key PST techniques, and was intrigued how these two approaches (ACT and PST) might complement each other, rather than being seen as separate and exclusive of each other. A cornerstone of applied sport psychology (Andersen, 2009), PST is born of a cognitive-behavioural approach to sport psychology (Turner, 2022) and as such, can be integrated within a broader cognitive-behavioural approach to service delivery. Although each technique is effective in isolation, they are typically combined in interventions to address the client’s needs and specific sport demands (Andersen, 2009). The most frequently used PST techniques (e.g., goal setting, self-talk, imagery, relaxation, and concentration techniques; Andersen, 2009) are discussed below.

*Goal setting* is widely applied in sport (see Williamson et al., 2024). Process goals are specific and can increase self-efficacy due to increased perception of control, and may have a greater impact on performance than performance and outcome goals (Williamson et al., 2024). However, open goals are exploratory and focused on challenging the athlete to see how well they can do, avoiding specific outcomes, may lead to optimal performances (Swann et al., 2017).

*Self-talk* refers to the internal running commentary of events as they happen that most of us have (also referred to as verbal thinking or self-directed verbalisations) (see Van Raalte et al., 2016). In sport, self-talk is considered a widely used and effective strategy for enhancing performance through both instructional and motivational statements (Van Raalte et al., 2016).

*Imagery* encourages the athlete to mentally practice their sport deliberately, focused on identifying and correcting poor performance or preparing for upcoming challenges. Often delivered alongside other psychological skills (Andersen, 2009), imagery engages sensory qualities and can evoke strong emotional states due, in part, to the link between imagery and perception (Holmes and Mathews, 2010). Many people experience automatic thoughts as unspoken words or as mental pictures or images (Beck, 2011). Hence, imagery is a form of cognition, viewed as containing meaning that is related to beliefs about oneself, others, and the world.

*Relaxation techniques* are integral to helping athletes maintain optimal arousal levels and effectively manage tension and psychological anxiety (Andersen, 2009). PST incorporates progressive relaxation, autogenic training, and biofeedback methods to reduce activation of the sympathetic nervous system and associated muscle tension (Andersen, 2009). However, of relevance to us is the use of centring, breathing exercises, and mindfulness in PST (Andersen, 2009).

*Concentration training* relates to an athlete’s ability to focus, switch attention, and disregard internal or external distractions; arguably one of the most crucial factors for athletes in sport (Moran, 2016). Concentration training helps the athlete focus their attention on the task at hand, limiting the likelihood of being affected by irrelevant external and internal stimuli (Wilson et al., 2006).

## The case

The client, Lucy (pseudonym), is an 11-year-old female figure skater. She competes in the lower levels of national competition. She liked the social aspect of the sport, and the friendships she made, as well as “achieving stuff.” When she started in the sport, aged 7, she “didn’t expect to get this far” but now had goals of competing at the highest levels, or becoming a coach. Age 6, at the start of the COVID-19 pandemic, she had received psychological support through the National Health Service’s Child and Adolescent Mental Health Service (CAMHS). This focused on general anxiety, comprising six counselling sessions, delivering parent-led CBT (covering skills like restructuring thoughts, distracting from thoughts, explaining fight and flight responses to stress). Although Lucy’s anxieties improved, she was now seeking sport-specific psychological support for performance-related anxiety. She found it difficult to apply previously learned strategies to her sport context.

## Needs analysis

Initial interactions with Lucy and her parents were like a dance (Tod and Eubank, 2020) as we collaboratively explored Lucy’s situation, and her understanding of it, prioritising the presenting problem and the focus of our work together (Keegan, 2016). Informed by an interpretative, constructivist, and person-centred approach, I felt questionnaires and measures were unhelpful, impersonal, and unable to represent Lucy’s inherently unique worldview and experiences (see Keegan, 2016). The primary needs analysis tool was conversation, informed by the Sport-Client Intake Protocol (SCIP; Taylor and Schnieder, 1992).

We identified performance-anxiety as the presenting problem, where anxiety had a negative impact on performance (see Multidimensional Anxiety Theory; Martens et al., 1990). Lucy would start feeling anxious the week before competition, resulting in her struggling with technical skills and crying a lot in training and on the day of competition. This was reflected in how she discussed feeling sick, her heart racing, hands shaking, her legs feeling like jelly, yawning, and feeling erratic. Here, negative thoughts (e.g., “something’s going to happen”) would tend to take over, where she was unable to “get out of [her] mind.” As a result, Lucy would become angry at herself. Reviewing Gardner and Moore (2004) Multi-Level Classification for Sport Psychology (MCS-SP), it seemed Lucy’s case aligned with performance dysfunction; her progress was slowing because of her thoughts, primarily caused by previous life events. Consequently, the primary focus of our work was to improve Lucy’s athletic performance (Gardner and Moore, 2004). Through the needs analysis, we agreed that our work would focus on supporting Lucy to better manage her performance anxiety.

## Case formulation

Taking an ACT approach, Lucy’s unhelpful thinking dominated her behaviour in training in a problematic way, which connected with the notion of cognitive fusion (Harris, 2019). Lucy’s thoughts were “hooking” her away from her desired way of training – *doing what matters* (Harris, 2019). Rather than changing Lucy’s thoughts like second wave CBTs (Young and Turner, 2023), which would add to her struggle, I felt we could explore strategies that would allow Lucy to accept her unhelpful thoughts about competition performance and feel happier and less distracted by these thoughts in the build-up to competition. In line with the construalist approach, I did not feel there was an “off the shelf,” ready-made intervention. Further, ACT encourages practitioners to continually develop new and varied strategies within interventions (Smout, 2012).

Given that PST can be effective with youth athletes (Thrower S. N. et al., 2024), exploring how ACT and PST could be integrated within this case seemed a worthy pursuit. However, little has been written about how SEP interventions can combine PST alongside popular third-wave CBTs, like ACT. Recently, Wood and Turner (2025a) conceptualised how ACT and PST might be integrated (outlined in Table 1). This current case study complements that work by outlining how PST and ACT *can* be integrated into an intervention, using PST techniques as a vehicle to introduce ACT processes when working with a youth athlete. Doing so, we hope to address calls for “how to” frameworks, contributing to resources for SEP practitioners working in youth sport (Thrower S. et al., 2024) while supporting neophyte SEP practitioners to establish a theoretical orientation for service delivery. Importantly, youth athletes have greatest knowledge of goal setting and mental imagery, whereas self-talk and relaxation appear less well explained (McCarthy et al., 2010). This is unsurprising given children’s active imagination and creativity. Imagery can support faster learning of new skills (Caliari, 2008), increase motivation (Hausenblas et al., 1999), and confidence (Callow and Waters, 2005). Goal setting encourages young athletes to identify their progress against performance standards (Thompson and Hilliard, 2021). Teaching concentration can increase youth athletes’ abilities to focus, as can self-talk, and linking the association between positive self-talk and performance in youth athletes is paramount (Hatzigeorgiadis et al., 2011).

**TABLE 1:** Aligning PST with ACT processes.

Psychological skill	Description	Related ACT processes
Imagery	Deliberate mental practice of sport performance	Defusion, acceptance, committed action
Goal setting	Related to the aim of an action and what an individual is trying to achieve	Values and committed action
Self-talk	A variety of techniques to refute negative consequences of troublesome/anxiety-provoking cognitions	Defusion, acceptance, and self-as-context
Relaxation	Help athletes maintain optimal arousal levels and manage psychological anxiety	Contacting with the present moment, self-as-context
Concentration	Focusing attention on the task at hand, limiting impact of external and internal stimuli	Defusion, values, committed action, contacting with the present moment

## Intervention plan, delivery, and monitoring

Written consent for the intervention was provided by Lucy’s parents. Verbal assent was gained from Lucy before the intervention formally commenced and when formally establishing and planning the intervention delivery.

In total, service delivery spanned 3 months, consisting of six sessions (roughly one every 2 weeks), varying in length from 50–60 min. Sessions followed a similar structure (e.g., recap and reflections on/since the last session, psychoeducation, experiential exercises, reflect and recap on session content) with some flexibility to meet Lucy’s needs, altering the focus, flow, and pace of sessions accordingly. Sessions were conducted online, via video call. The virtual delivery of CBTs has proven to be as effective as in-person therapy (Thomas et al., 2021) in the sporting domain (Price et al., 2022). In line with Payne et al. (2020) I established therapeutic boundaries in the virtual domain by: maintaining a neutral and consistent background to calls; assuring Lucy I was the only one in the room, maintaining confidentiality; ensuring my screen was large enough to see Lucy’s facial expressions during sessions; and established a strong relationship by being active in discussions, using more open and directive questions. Outlining the confidentiality that informed our work, Lucy was assured that any discussion could be had without her parents’ presence, if she preferred. This was not the case, with Lucy’s parents attending all sessions.

**FIGURE 1:**
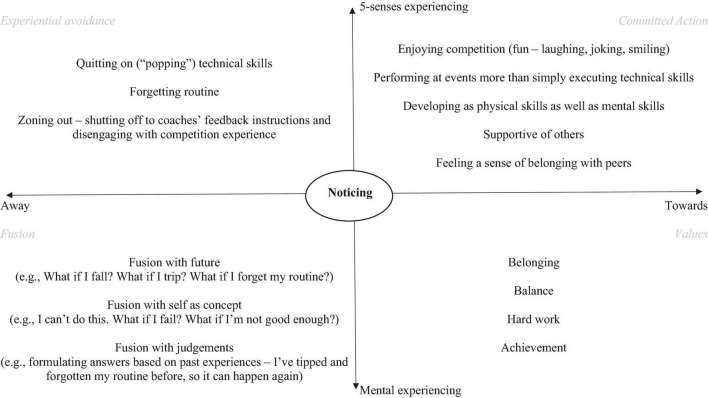
Lucy’s completed ACT matrix.

I used the ACT matrix (Polk and Schoendorff, 2014) throughout the intervention to conceptualise Lucy’s experiences (Figure 1) and frame the strategies we discussed to target the core processes of psychological flexibility (Harris, 2019). Previous experience had highlighted how confusing some aspects of the ACT triflex can be, and the ACT matrix had helped in this regard.

### Exploring Lucy’s strengths, values, and goals

With no right place to start ACT interventions (Wood and Turner, 2025b), our work began with a performance profiling task (Butler and Hardy, 1992). This is an effective place to start goal setting – a cornerstone of PST (Andersen, 2009) – with youth athletes (Foster et al., 2016). I adapted the task to initiate key ACT processes, specifically values clarification and committed action. This task enabled Lucy to actively participate in her own performance analysis (Weston et al., 2011), increasing her self-awareness (Castillo and Chow, 2020) and offering insight into her psychological (e.g., commitment, discipline, and confidence) and physical characteristics (e.g., flexibility, power, stamina, and technical skills). This was a useful task to build rapport with Lucy, and utilising personal construct theory (Kelly, 1995) gave insight to her thoughts about her sport and perceived abilities. This exercise also asked Lucy to consider what was important to her in her sport (i.e., values) by considering the athlete she wanted to be. Here, unlike a traditional values task, where the athlete is presented with a series of values to identify, we framed exploration of values around the profiling task.

When discussing goals, Lucy started by sharing her outcome goals for the season. I encouraged Lucy to shift focus from outcome-focused goals that are heavily focused on technique and instead focus on the broader, process-oriented behavioural intentions, which she arguably had more autonomy over. This is consistent with ACT’s committed action and the process of enacting values through behaviour (Hayes et al., 2012). For Lucy, this meant reframing goals as actions such as “be disciplined on competition days by executing my pre-performance routines” or “deliver a routine that performs to the music,” embedding psychological flexibility.

### Visualising the athlete Lucy wanted to be

To help Lucy overcome some of her performance anxieties, we addressed any confidence issues that she might have been experiencing using imagery, an established PST tool that encourages athletes to mentally rehearse their sport (Saint-Martin et al., 2020). Lucy shared that she had previously tried imagery, taught to her by her coach, but had struggled to connect with it as a skill. However, she discussed having dreams when she slept and liked learning skills by watching others execute them at training. We started by having Lucy watch videos of herself executing skills in training or competing. We set out to do this as a homework task three times per week.

Using the PETTLEP model (Holmes and Collins, 2001) to structure imagery practice, I emphasised the importance of making the imagery feel as realistic as possible, incorporating sensory qualities (Hackmann et al., 2011). Importantly, we linked this imagery practice to ACT’s concept of committed action (Hayes, 2016). Here, the goal was to have Lucy visualise the traits she felt were important. For example, Lucy focused on her performance to the music, the result of her hard work (e.g., flexibility in technical skills that set her apart from her competitors rather than a perfect routine where she successfully executed every skill) and committing to performance and technical skills. In this way, we reinforced committed action, anchoring psychological skills in a value-consistent framework.

### Handling unhelpful self-talk

In PST, self-talk captures the techniques used by the athlete to handle troublesome, anxiety-inducing cognitions (Andersen, 2009). Rather than following the traditional PST self-talk strategies that aim to replace negative thoughts with positive affirmations (Andersen, 2009), I introduced cognitive defusion techniques from ACT. This shift reflected ACT’s third-wave orientation, which emphasises altering the function – not the form – of internal experiences (Hayes et al., 2012). I was mindful of keeping discussions around this part of the hexaflex model simple for Lucy and focused on making defusion experiential, metaphor-driven, and accessible. First, we discussed how she would normally react to unhelpful thoughts. Lucy explained how she would disagree with the thought and tell herself the opposite (e.g., telling herself she could do something if she thought she couldn’t). We explored how effective this typically was for her. Lucy explained it rarely worked, leading to more frustration and anxiety. Lucy’s habit of arguing with her thoughts provided a natural entry point for the “tug of war” metaphor, illustrating how resistance often intensified distress, and proposed the new approach of “dropping the rope.”

ACT assumes that unhelpful self-talk negatively affects performance when cognitive fusion occurs – where an event and our cognitions about it become inseparable. This means we respond to thoughts as though they are literal truths, restricting actions towards what is important (Ruiz et al., 2023). Consequently, defusion strategies encourage athletes to experience thought and feelings as mental events, rather than reflective of reality (Ruiz et al., 2023). Creating distance between the individual and thoughts allows for flexibility in thinking and lessens the power the unhelpful thought has on emotions and behaviours (Harris, 2019). There is no agreed-upon categorisation of defusion processes linked with possible interventions and the ACT literature is yet to evidence the effectiveness of one approach to defusion over another (see Ruiz et al., 2023). I discussed three routes to defusion with Lucy. First, we discussed singing her thoughts (in her head) to a tune (like happy birthday). This promotes defusion by shifting the stimulus control from the word’s meanings to their formal properties. Second, we discussed visualising the thought as a character – the self-doubt monster. Attaching physical properties to thoughts in this way establishes spatial distance between the athlete and their thoughts (Assaz et al., 2023). Third, we discussed “thanks brain” as a response to thoughts, encouraging Lucy to separate the thought (her brain) from herself as an individual (Lucy). Initially, Lucy connected with the idea of singing her thoughts. However, on reflection she found the repetitive exposure to the thought unhelpful, over time. We revisited the “thanks brain” response, encouraging the use of sarcasm, and Lucy connected better with this approach. This reinforces my functional and client-led approach to the intervention.

### Staying present

To further support psychological flexibility during performance, I then discussed connecting with the present moment when negative self-talk became distracting or overwhelming. While PST might incorporate progressive muscle relaxation to increase relaxation (Talha, 2023), ACT encourages breathing exercises and sense-checking exercises to encourage a focus on here-and-now experiences. These helped Lucy engage in the physical world around her, rather than the thoughts in her head. Lucy connected with the concept of “sense-checking” – noticing what she could see, hear, feel, touch – in each moment. We also discussed the colourful breathing exercise (Perry, 2020), engaging multiple senses (i.e., listening to and feeling breaths, while visualising colours) with breathwork. These techniques supported Lucy’s attentional control, allowing her to shift focus from internal distraction to the task at hand (Moran, 2016) and managing anxiety in the moment with the aim of enhancing her performance (Muangnapoe et al., 2016; Wilson et al., 2006).

## Evaluation and outcomes of the intervention

The intervention prioritised the ACT processes of committed action, cognitive defusion, and contact with the present moment. These were chosen for their practical accessibility, developmental appropriateness, and close alignment with Lucy’s performance issues. While ACT includes six interrelated processes, some – such as self-as-context – were not central in this intervention. Instead, the intervention was guided by ACT’s overarching aim to increase psychological flexibility – the ability to take effective, values-guided action in the presence of challenging internal experiences – to help Lucy manage her performance anxiety. The aim of this article is to demonstrate how ACT can be integrated with PST.

Systematic monitoring and evaluation of SPP’s work is critical to assessing their service delivery (Keegan, 2016). ACT interventions embed evaluation throughout with constant discussions around treatment goals (Hayes et al., 2004). Informally, we reviewed our work together following experiential exercises, throughout, and at the end of the intervention. Below, we present some reflections from the client, which we feel generate insight into Lucy’s experiences of this intervention. Reflections were captured using prompts from Hartley (2020), combined with feedback generated using a consultant evaluation form (see Partington and Orlick, 1987). Of course, we are mindful that there are individual difference factors impacting the perceived effectiveness of this intervention. Lucy’s time and level in the sport, her previous struggles and experiences of how her nervousness had impacted her performance in sport, the influence of her parents given her age, and her initial predisposition to psychological practice may have positively favoured the intervention process.

### What progress do you feel you have made during our work together?

Lucy thought we had made progress. She felt she was learning to calm herself when she became stressed, was feeling less anxious at competitions (meaning she was able to perform better in these moments), and was feeling more confident in her abilities, which was an unexpected outcome. Lucy’s parents both agreed that Lucy had made progress through our work together. They felt that previous counselling sessions covered skills that Lucy had struggled to apply to her sport context. In this sense, they felt part of Lucy’s progress was because strategies were contextualised to sport. They felt that my knowledge of her sport also helped Lucy relate to me and build confidence in me, which helped build our working relationship.

### To what extent have we achieved the goals of the delivery service?

Lucy thought we had achieved the goals of our work together. She felt better prepared for competitions and felt more able to handle her anxieties in the built up to, and on the day of, competition. Her parents discussed how our work together gave Lucy an anchor for stressful times – some structure when things became a little overwhelming.

### What would you change about how we have worked together?

Given Lucy’s responses to the above questions, she did not feel there was anything she would have changed in our work together. Lucy’s parents agreed. They attended every session with Lucy, providing additional support if Lucy struggled to understand a question or strategy we were discussing. I was content with this and outlined at the start that I was happy to take Lucy’s lead and have them present throughout the intervention if she felt more comfortable with that. At this stage in the intervention, they asked if I thought they should have withdrawn their support as the intervention developed. It was interesting to have this question returned, asking for my views. Lucy’s parents were great in sessions, letting Lucy express herself in sessions, supporting, but not overpowering discussions.

## Practitioner reflections and lessons learned

This section presents reflections that highlights the role of the sport psychologist when working with youth athletes and valuing the importance of PST in sport psychology. I hope these highlight some key messages to inform best practice for (future) effective service delivery (Knowles et al., 2007).

### Working with youth athletes

I have often heard peers share how they “don’t work with youth athletes.” While it is important to understand your strengths and limitations of practice as an applied professional to ensure you are operating within the practitioner’s competency (BPS, 2018), I am often shocked that some are comfortable to exclude such a large athlete population from their work. That is not to dismiss that working with this client group can be challenging. Away from the safeguarding concerns for some practitioners, this case made me reflect on the perceived stress of “getting it right” for this young athlete. Of course, I strive to “do no harm” in my practice and protect the mental health of every athlete I work with, regardless of age or standard. However, this case brought a different sense of perceived responsibility. The fact that parents, like Lucy’s, trust us with facilitating positive sporting experiences for their children should not be underappreciated. Of course, without focusing too heavily on an all-too-common debate in psychology (see BBC, 2021), this clearly highlights the importance of qualified practitioners operating within applied sport psychology (see Gilmore et al., 2018).

ACT, and its associated components, has proven helpful for adolescents (i.e., those aged 11–17 years old) experiencing obsessive-compulsive disorder (Armstrong, 2012), autism spectrum and learning disorders (Cook, 2009), depression (Hayes et al., 2011; Livheim et al., 2015), anorexia (Heffner et al., 2002), chronic pain (Wicksell et al., 2005), and post-traumatic stress disorder (Woidneck et al., 2014). Additionally, participants in several studies have shown ACT can increase mindfulness (Livheim et al., 2015), psychological flexibility (Cook, 2009), values-based living and congruence (Wicksell et al., 2005), while reducing avoidance and fusion (Szabo, 2019) in adolescent groups. Given this growing evidence-base, I was comfortable using ACT with Lucy. One key aspect of delivering CBTs to young clients is maintaining a developmentally appropriate manner to delivery to enhance motivation and engagement in therapy (see McLachlan et al., 2016).

When reflecting with Lucy’s parents at the end of the intervention, Lucy’s dad commented on how he had noticed how my use of questioning guided Lucy where I wanted the conversation to go without directly telling her. This was not consciously done, perhaps highlighting how instinctive this had become for me in my work with youth athletes. Give this, it is easy to overlook the importance of Socratic questioning – best described as systematic questioning and inductive reasoning that attenuates cognitive misinterpretations and other inductive or deductive errors (McLachlan et al., 2016) – to help clients discover answers and develop critical thinking skills. Of course, there were times where this style of questioning required more specific prompts to progress the conversation, and Lucy’s parents facilitated some discussion if my question needed breaking down, or modifying, for Lucy to understand.

### Valuing the importance of PST

This case study highlights the important role of PST in applied sport psychology. During my training for Chartered Psychologist status, I was encouraged, without much justification, to move “beyond’ PST in my service delivery. I started to integrate PST within my applied work when working with athletes during the COVID-19 pandemic. One prominent example is the use of imagery, which seemed a great way of encouraging athletes to stay connected to their sport setting when training facilities were closed. Reconnecting with these skills helped me understand, as highlighted by this case study, that encouraging practitioners to move away from PST is the result of not thinking deeply enough about the value of PST to applied practice. PST is derived from CBT, which generates endless possibilities of applying PST alongside broader CBT approaches, as demonstrated here. Importantly, taking this work alongside Munnik et al. (2023) and Wood and Turner (2025a), the CBT orientation of the SEP practitioner (e.g., ACT, REBT, etc.) will shape how the PST techniques are used. Importantly, PST is not simply a collection of standalone techniques without theoretical grounding. PST is a vital component of the CBT canon and, when applied with ACT, can offer effective ways to operationalise strategies that can help athletes to achieve in performance settings. Further, given the effectiveness of PST with youth athletes, and the likelihood that most athletes and SEP practitioners are familiar with PST, practitioners might consider the use of PST as a Trojan horse for integrating PST with other CBT approaches with the aim of developing effective professional practice.

## Conclusion

To conclude, Lucy reported feeling less anxious, even though she still experienced thoughts and feelings associated with anxiety. The aim of our work was not to remove or restructure Lucy’s thoughts, but help her “sit” with them, defusing them and working through the associated challenges so she could move towards what was important for her (i.e., values) and the athlete she wanted to be (i.e., committed action). We hope we have demonstrated transparency in reporting this case study. In navigating different service delivery philosophies and approaches, PST might offer practitioners in SEP settings a familiar and safe option with which other CBTs can be blended. Complementing the work of Wood and Turner (2025a), we hope to further highlight the similarities across both ACT and PST. Rather than provide a manualised protocol of how interventions *should* blend PST with ACT, we demonstrate how an intervention *might* integrate ACT into PST. By focusing on youth athletes and evidencing a way in which ACT can be delivered with children and young people in sport settings, this case study adds to the wider literature in psychotherapy in sport. We hope to encourage SEP practitioners to be inclusive and increase confidence for those who are unsure about working with young populations, as well as those new to applying ACT to their interventions.

## Data Availability

The data presented in this article are not readily available because they contain case notes from applied practice. Requests to access the data set should be directed to SW, s.wood@mmu.ac.uk.
